# Reusing Kaolin Residue from the Mining Industry to Produce PCL-Based Composites: Accelerating the Crystallization Process and Improving Mechanical Properties

**DOI:** 10.3390/ijms26104632

**Published:** 2025-05-13

**Authors:** Carlos Bruno Barreto Luna, Jessika Andrade dos Santos Nogueira, José Vinícius Melo Barreto, Elieber Barros Bezerra, Fabiano Santana da Silva, Lorena Vanessa Medeiros Dantas, Renate Maria Ramos Wellen, Edcleide Maria Araújo

**Affiliations:** 1 Academic Unit of Materials Engineering, Federal University of Campina Grande, Av. Aprígio Veloso, 882-Bodocongó, Campina Grande 58429-900, PB, Brazil; jessikandrde@gmail.com (J.A.d.S.N.); elieberbarros@hotmail.com (E.B.B.); sunsolaris8@hotmail.com (F.S.d.S.); lorena9dantas@gmail.com (L.V.M.D.); edcleidemaraujo@gmail.com (E.M.A.); 2 Department of Materials Engineering, Federal University of Paraíba, Cidade Universitária, João Pessoa 58051-900, PB, Brazil; viniciusbarreto89@hotmail.com (J.V.M.B.); wellen.renate@gmail.com (R.M.R.W.)

**Keywords:** PCL, kaolin residue, composites, phase transition, activation energies, kinetics

## Abstract

The impact of adding 1%, 3%, and 5% by mass of kaolin residue (KR) was investigated regarding the mechanical, thermomechanical, and morphological properties, as well as the non-isothermal crystallization and melting kinetics of poly(ε-caprolactone) (PCL). The processing to obtain the PCL/KR composites was carried out through extrusion in a twin-screw extruder, followed by injection molding. This study investigated the events of first melting, fusion crystallization, and second melting using differential scanning calorimetry (DSC), with heating rates ranging from 5 to 25 °C/min. Additionally, models for the expanded Prout–Tompkins equation (BNA), the nth-order reaction with m-power autocatalysis by product (Cnm), and the Sestak and Berggren equation (SB) were tested. The PCL/KR composites exhibited an increase in the elastic modulus and the heat deflection temperature (HDT) compared to the pure PCL. Furthermore, high ductility was observed, as evidenced by the impact strength and elongation at break. The good distribution of KR in the PCL matrix was confirmed by scanning electron microscopy (SEM), which contributed to a more efficient crystallization process. The increase in KR content in the PCL matrix shifted the crystallization sigmoids to higher temperatures, acting as a nucleating agent, which reduced the energy barriers and increased the crystallization temperature by up to 5 °C. The melting events did not show significant changes with the addition of the KR. The results are important for the plastics processing industry, mainly due to the opportunity to add value to the waste and use it as an additive.

## 1. Introduction

Poly(ε-caprolactone) (PCL) is a biodegradable semicrystalline polyester with a regular structure, widely utilized in engineering applications such as packaging, biomaterials, agriculture, and additive manufacturing [1,2,3,4,5]. Notably, PCL has been employed as a biodegradable packaging material, as it can be decomposed by microorganisms [6,7]. PCL exhibits promising physical, chemical, and biological properties for technological applications, offering excellent processability and flexibility, along with a glass transition temperature (T_g_) of −60 °C [8,9]. These characteristics make it a valuable material for eco-friendly and innovative solutions [10,11]. However, PCL undergoes a slow solidification process, particularly after being processed in the molten state and subsequently cooling to ambient temperature. As a result, nucleating agents have been explored as a means of accelerating the polymer crystallization process, generating prospects for reusing mineral filler residues from the mining industry to enhance the crystallization cycle [12]. In this context, kaolin waste emerges as an alternative to be investigated for its technological potential as a filler to accelerate polymers crystallization.

Kaolin is a mineral composed of hydrated aluminum silicates, primarily kaolinite and halloysite. While kaolinite (Al_2_O_3_·2SiO_2_·2H_2_O) is the principal component of kaolin, other elements, beyond aluminum, hydrogen, oxygen, and silicon, may be present in more complex forms [13]. Kaolin holds significant industrial importance due to its properties, including chemical inertness across a broad pH range, white coloration, excellent covering ability, non-abrasiveness, low thermal and electrical conductivity, and low cost [14]. The initial industrial application of kaolin was in the production of ceramic materials and porcelain. It was only from 1920 onward that kaolin began to be utilized in the paper industry [15]. Other applications include the rubber [16], pesticide [17], and fertilizer [18] industries. Kaolin residue (KR), a byproduct of the paper industry, can be reintegrated into the production chain and applied in the plastics sector to meet various engineering demands. Additionally, it contributes to reducing industrial costs, such as serving as a nucleating agent during the molding cycle of polymer products.

The incorporation of mineral fillers into the PCL matrix can enhance its mechanical, thermomechanical, thermal, and crystallization kinetics performance, thereby improving its ability to meet the demands of the plastics technology sector while also reducing the production cycle during molding [19,20]. However, the type of processing and the screw profile in the extruder can influence the final properties of the product, as they affect the degree of distribution and dispersion of the mineral filler in the PCL matrix. This directly reflects on the crystallization kinetics of PCL. Inorganic fillers in the PCL matrix can promote crystallization, as mineral particles serve as nucleation sites, lowering the energy required to solidify the material and facilitating the premature initiation of crystallization at higher temperatures [21,22,23,24].

Paula et al. [25] synthesized PCL/zinc oxide (ZnO) films through solvent dissolution, employing ZnO concentrations ranging from 0.5% to 5%. The authors observed that PCL exhibits a crystallization temperature of 20.4 °C, while the incorporation of ZnO accelerated this process, shifting the crystallization temperature to a range of 20.7 °C to 23.5 °C, indicating a nucleating effect. In the study conducted by Lanfranconi et al. [26], PCL nanocomposites containing 2.5%, 5.0%, and 7.5% by mass of two organo-modified clays were prepared through melt intercalation. The results demonstrated that the presence of clay influenced the isothermal crystallization process of the nanocomposites. The induction time and overall crystallization rate were highly dependent on the degree of clay dispersion. Nanocomposites with low levels of clay dispersion in the PCL matrix exhibited shorter induction times, accelerated crystallization rates, and higher Avrami rate constants. However, the addition of 5% clay promoted the formation of agglomerates, and harmful effects on the crystallization process were observed. Filho et al. [27] prepared poly(ε-caprolactone) (PCL) nanocomposites reinforced with montmorillonite clay (MMT) (3 and 5% by mass) using melt processing. The torque rheometry analysis indicated a slight increase in the viscosity of the PCL/MMT nanocomposites compared to pure PCL. The properties of elastic modulus and heat deflection temperature (HDT) showed an increase, suggesting higher rigidity and thermomechanical stability. The nanocomposites exhibited ductile behavior under tensile stress, with elongation exceeding 350%. Regarding the crystallization process, the clay acted as a nucleating agent, accelerating crystallization.

There are several studies that have been conducted on the thermal properties and crystallization kinetics of PCL with the addition of nanowhiskers and microfibrillated cellulose [28], graphite nanoplatelets [29], niobium pentoxide and alumina [30], sepiolite [31], and natural fibers [32], all of which suggest the potential for adjusting the crystallization process of PCL. However, these additives are high-cost materials, which drives the search for new alternatives derived from industrial waste to accelerate this process. Kaolin, an abundant and low-cost raw material, particularly when sourced from mining industry residues, emerges as a promising alternative. Given the limited literature on the effect of kaolin residue incorporation on the mechanical properties and crystallization of PCL, there is a need for investigations into the technological potential of this waste. This could enable the reintroduction of kaolin residue into the plastic technology production chain, particularly as an additive.

Therefore, the present investigation aimed to produce PCL composites based on kaolin residue (KR), with the objective of investigating mechanical properties, thermomechanical properties, crystallization, and morphology. The melting and crystallization of PCL and PCL/KR composites were monitored using differential scanning calorimetry (DSC) to assess the effect of the KR filler on phase transitions, varying the KR content from 1 to 5% by mass. The crystallization kinetics were investigated using methods based on the Prout–Tompkins (BNA) equation, the nth-order reaction with m-power autocatalysis by product (Cnm), and the Sestak–Berggren (SB) model. This study reports the activation energies of the melting processes, determined by the Friedman method, based on the effect of the KR filler. Regarding crystallization’s activation energies, the crystallization mechanism’s characteristic functions were evaluated using experimental data and applied models. This work on PCL/KR composites can clarify the impact of KR on the crystallization process, promoting energy savings. Furthermore, it contributes to the advancement of kaolin residue reutilization in the production of PCL composites, guiding the development of sustainable materials.

## 2. Results and Discussion

### 2.1. Impact Strength

**Figure 1:**
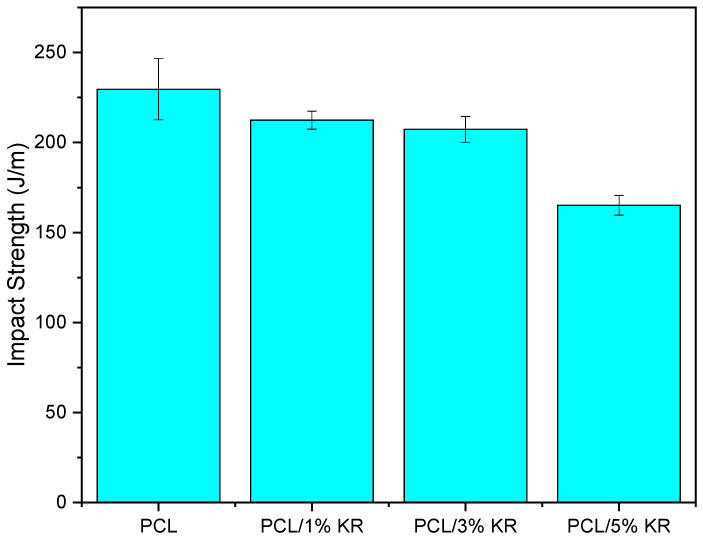
Impact strength of PCL and PLA/KR composites, with different KR concentrations.

In Figure 1, the impact strength of PCL and the PCL/KR composites with KR contents ranging from 1 to 5% is presented. The pure PCL exhibits a typical tough material behavior at room temperature, with an impact strength of approximately 230 J/m. The PCL/1% KR and PCL/3% KR composites show a reduction in impact strength of 7.5% and 9.7%, respectively, compared to the pure PCL. However, this reduction is not drastic, considering that a fraction of the PCL is replaced by kaolin residue (KR). Additionally, the impact strength of the PCL/1% KR and PCL/3% KR composites is similar, as the values fall within the experimental error margin. In contrast, the impact behavior decreases more severely for the PCL/5% KR composite, with a 28.2% decline compared to the PCL. Since kaolin residue is a rigid mineral filler, there is a tendency to embrittle the PCL. The literature [33,34] indicates that mineral fillers concentrate stresses in ductile thermoplastic matrices, restrict molecular mobility, and reduce ductility. This results in premature fracture, leading to a lower energy dissipation capacity during the impact test.

**Figure 2:**
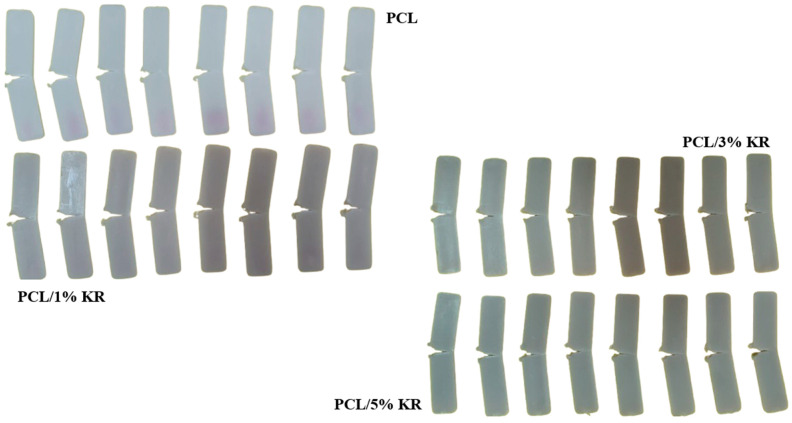
Test specimens after impact testing for PCL and PCL/KR composites.

The PCL/KR composites are interesting for applications that require high impact resistance at ambient temperature, considering that the lowest performance is for PCL/5% KR with 165.1 J/m. For example, this result of 165.1 J/m surpasses polyethylene (PE) [35,36], polypropylene (PP) [37,38], and polyamide 6 (PA6) [39,40]. Furthermore, the reuse of kaolin waste as an additive for PCL helps minimize environmental pollution, focusing on reintroduction into the plastic transformation production chain and the production of sustainable materials. Figure 2 shows the behavior of the samples after the impact test with the 5.5 J pendulum, suggesting a “P”-type fracture, according to the ASTM D256 standard.

### 2.2. Tensile Properties

**Figure 3:**
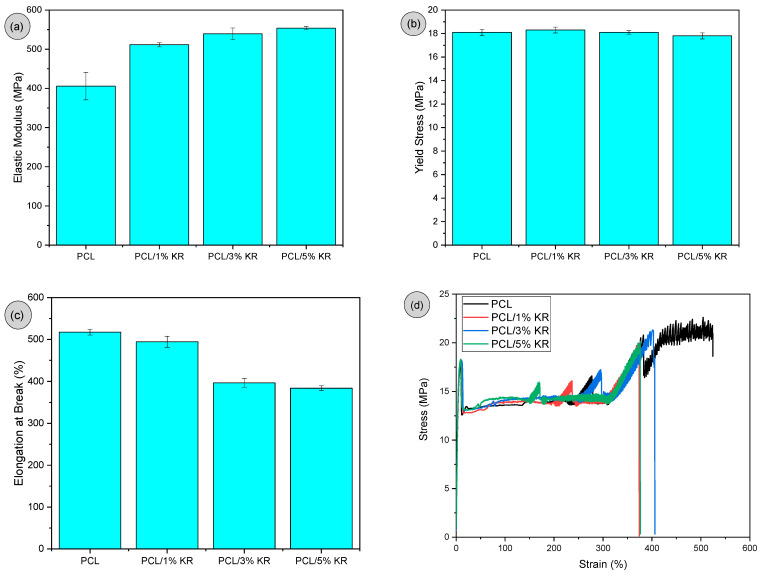
Mechanical properties under tensile for pure PCL and PCL/KR composites, for (**a**) elastic modulus; (**b**) yield strength; (**c**) elongation at break; (**d**) stress vs. strain curves.

The mechanical properties under tensile of elastic modulus, tensile strength, and elongation at break for pure PCL and the composites are presented in Figure 3a–c. The stress vs. strain curves are shown in Figure 3d. In Figure 3a, it was observed that the pure PCL exhibited an elastic modulus of around 405 MPa, while the addition of 1% KR increased it by 26%. By increasing the KR content to 3% and 5% in the PCL matrix, the elastic modulus continued to increase, with gains of 32.9% and 36.4%, respectively. The PCL/KR composites, regardless of the KR concentration, are stiffer compared to the pure PCL. Therefore, the incorporation of kaolin waste into PCL restricted molecular mobility, leading to an increase in stiffness. However, in the 3% and 5% KR compositions investigated, the elastic modulus results are quite similar, suggesting comparable behaviors for this property. As demonstrated in the literature [41,42], mineral fillers are stiffer materials compared to the polymeric matrix, and their addition generally increases the composite’s stiffness. This happens because the particles of mineral fillers dispersed in the polymeric matrix hinder molecular mobility, resulting in lower performance of deformation.

In Figure 3b, it can be observed that the pure PCL exhibited a maximum yield stress of around 18.1 MPa, which is consistent with the values reported in the literature [43]. The influence of KR concentration on PCL is minimal for the yield stress, meaning there was no reinforcement effect. The KR contents ranging from 1% to 5% in the PCL/KR composites provided results similar to pure PCL, with values fluctuating between 17.8 and 18.5 MPa. Therefore, kaolin waste behaved as a non-reinforcing filler in PCL, improving the elastic modulus while maintaining the yield stress.

In Figure 3c, the elongation at break of the pure PCL was quite high, with deformation exceeding 500%. This is typical of a polymer with a high degree of ductility and high plastic deformation. There was a tendency for elongation at break to decline in the PCL/KR composites, especially with higher amounts of KR. The incorporation of 1% KR into the PCL does not seem to significantly influence the elongation at break (~495%), considering the result is close to the experimental error margin. The decline in elongation at break was more pronounced with KR contents of 3% and 5% in the PCL matrix, with values of 396.9% and 384.1%, respectively. This indicates that a higher amount of kaolin waste in the PCL inhibited the plastic deformation mechanism, leading to premature failure during the tensile test. The distribution of kaolin residue in the PCL chain, especially at 3% and 5%, promoted greater obstacles to molecular movement, making it difficult for the chains to slide and, consequently, reducing the degree of plastic deformation [44,45]. However, the PCL/3% KR and PCL/5% KR composites maintained a high level of plastic deformation, confirming the trend observed in impact strength. As shown in Figure 3d, the degree of ductility was quite high for both the PCL and the PCL/KR composites, suggesting that before fracture, there was significant plastic deformation.

### 2.3. Shore D Hardness

**Figure 4:**
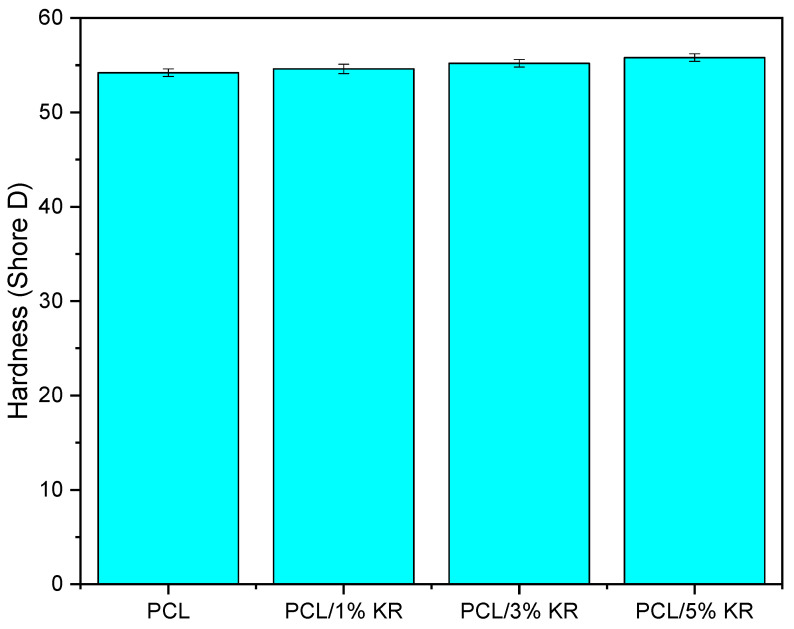
Shore D hardness of PCL and PCL/KR composites, with different KR contents.

The influence of the addition of KR on the Shore D hardness of PCL is also investigated, as shown in Figure 4. The pure PCL exhibits a Shore D hardness of 54.2, a value close to that reported by Garcia et al. [46]. It is observed that the incorporation of kaolin residue into the PCL does not result in a significant increase in the Shore D hardness of the composites, with only slight fluctuations between 54.6 and 55.8 Shore D. In this case, the use of low KR concentrations in the PCL is not sufficient to increase the resistance to penetration, only maintaining this property.

### 2.4. Heat Deflection Temperature (HDT)

**Figure 5:**
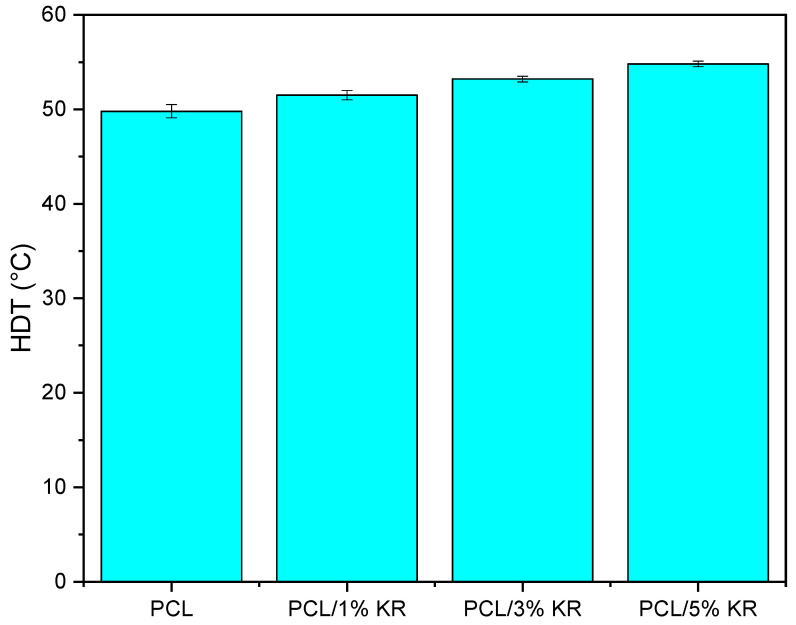
HDT behavior for pure PCL and PCL/KR composites, as a function of the amount of kaolin residue.

Figure 5 demonstrates the behavior of the heat deflection temperature (HDT) for the pure PCL and the PCL/KR composites, aiming to evaluate the structural behavior under the effect of temperature and mechanical load. The HDT of the PCL is 54.2 °C, a low value due to its flexible behavior, as observed in the elongation at break. The kaolin residue contributes to a slight increase in the HDT of the PCL/KR composites, confirming the trend observed in the elastic modulus. A progressive increase in HDT is observed with the addition of KR to the PCL matrix. For example, the PCL/5% KR composite shows the highest performance, with a gain of around 4%, although this is not considered a significant improvement.

### 2.5. The Impact Fracture Surface of PCL and the PCL/KR Composites

**Figure 6:**
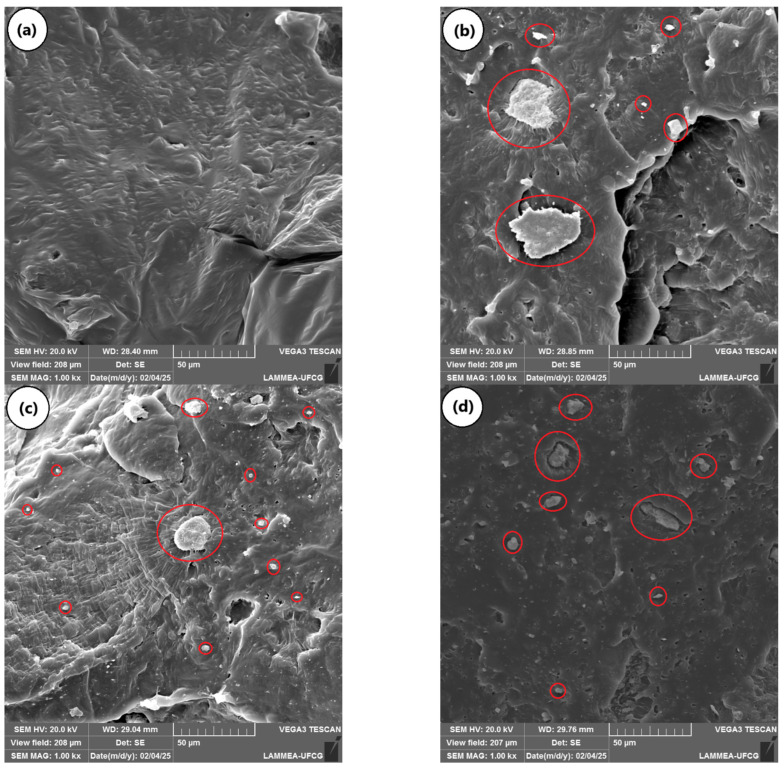
Morphology obtained by SEM with 1000× magnification, for (**a**) PCL; (**b**) PCL/1% KR; (**c**) PCL/3% KR; (**d**) PCL/5% KR. Kaolin residue particles marked in the red circles.

Figure 6a–d show micrographs obtained through scanning electron microscopy (SEM) of the fracture surface of the PCL and the PCL/KR composites. In Figure 6a, a typical fracture of a ductile polymer was observed for the PCL, with plastic deformation and surface roughness, suggesting resistance to crack propagation. The morphology of the PCL supports the high impact strength value, as presented earlier. Regarding the PCL/KR composites, a good distribution of kaolin waste in the PCL matrix was observed, which likely contributed to the good mechanical results. Furthermore, there was heterogeneity in the particle size of the KR, with some large particles and other small particles (see red circles). The PCL/1% KR and PCL/3% KR composites exhibited a rougher fracture surface compared to the PCL/5% KR, indicating higher ductility. Apparently, the PCL/5% KR composite had a greater number of voids, suggesting that KR particles were pulled out from the PCL during the impact test, which aligns with the lower impact performance. In Figure 6d, it can be seen that the PCL/5% KR interfacial region presents some KR particles with low wettability with PCL, which results in higher stress concentration during the mechanical test. Low interfacial resistances in polymeric composites are generally identified on fracture surfaces by the presence of voids, associated with particle pull-out, caused by crack propagation through the interfacial region [47,48,49].

### 2.6. DSC Measurements

**Figure 7:**
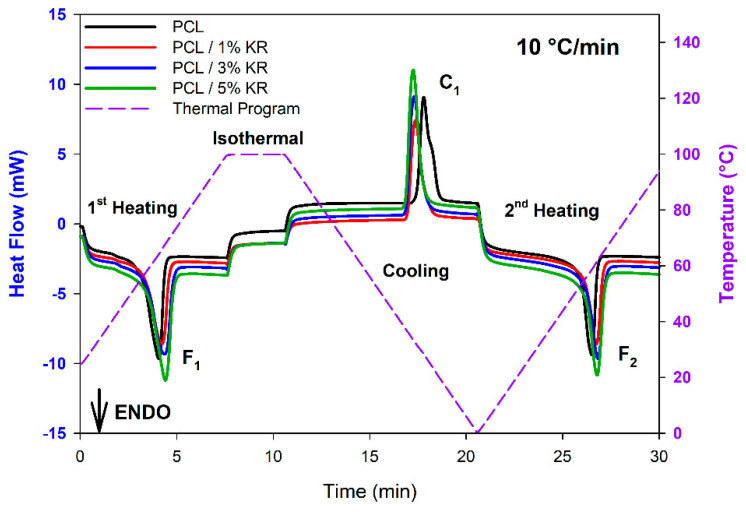
DSC scans showing heating, cooling, and reheating cycles at 10 °C/min, with the applied thermal program represented by the purple line. The investigated phase transitions, F_1_, C_1_, and F_2_, and the corresponding composites are indicated.

The phase transitions and thermal properties of the PCL and PCL/KR composites were analyzed using differential scanning calorimetry (DSC). Figure 7 shows a DSC scan of the applied thermal program illustrated as a dotted purple line for 10 °C/min, while the other heating rates are presented in Supplementary Material (Figure S1). The phase transitions investigated are labeled as follows: F_1_ (first melting), C_1_ (crystallization upon melting), and F_2_ (second melting). The cooling cycle shows that the addition of the kaolin residue (KR) filler promoted crystallization at higher temperatures compared to the pure PCL, acting as a nucleating agent. At the same time, there was a slight reduction in the time required to crystallize the PCL/KR composites. The acceleration of crystallization can reduce the time required for polymer solidification, which, in turn, speeds up the manufacturing process. This is particularly advantageous in industries that require large-scale production, such as the plastic processing industry. The effect of the filler will be further examined through curve integration and parameter evaluation in the section on melt crystallization measurements.

### 2.7. Melt Crystallization (C_1_) Measurements

**Figure 8:**
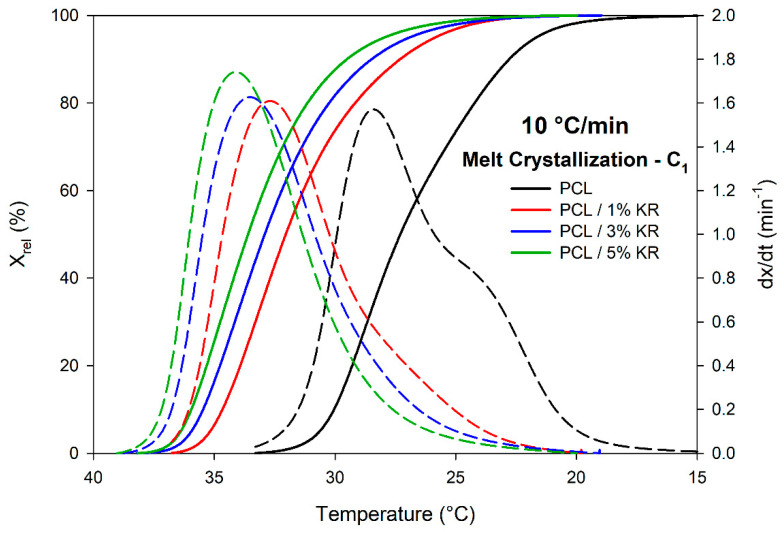
Relative crystallinity (solid line) and crystallization rate (dotted line) as temperature function. Composites and cooling rates indicated.

The relative crystallinity (X_rel_) and crystallization rate (dx/dt) as functions of temperature for PCL and PCL/KR composites under a cooling ramp of 10 °C/min are shown in Figure 8. The other curves at rates of 5 °C/min, 15 °C/min, 20 °C/min, and 25 °C/min can be found in the Supplementary Material (Figure S2). Additionally, the thermal parameters of the maximum crystallization rate (c_max_), crystallization onset temperature (T_1C_), crystallization peak temperature (T_PC_), crystallization completion temperature (T_2C_), and process enthalpy (ΔH) can be found in Table 1.

As shown in Figure 8, the PCL and PCL/KR samples exhibited a sigmoidal profile, indicating a phase transition without discontinuities, a characteristic behavior of polymers. The crystallization rate (dx/dt) displayed a bell-shaped curve. Additionally, the initial increase in the crystallization rate is associated with nucleation and primary crystallization, reaching c_max_ before decreasing, as the process transitions to secondary crystallization [50]. For higher cooling rates, the sigmoidal curves shifted to lower temperatures due to the time effect. At higher heating rates, nucleation takes less time, and crystal growth occurs at lower temperatures [51]. Overall, the maximum crystallization rates increased with higher cooling rates, as evidenced by the peaks of the dx/dt curves.

Regarding the addition of KR to the PCL matrix, it was observed that the filler acted as a nucleating agent, promoting a shift of the crystallization sigmoid to higher temperatures [32]. At a 10 °C/min cooling rate, the crystallization onset temperatures (T_1C_) for the PCL and PCL/KR (1%, 3%, and 5%) were 32.2, 35.6, 36.2, and 36.5 °C, respectively. The increase in the KR content in the PCL resulted in higher T_1C_ and T_PC_ temperatures. In processes such as injection molding, accelerating the crystallization of PCL/KR composites can improve process efficiency by reducing the production cycle time. This is beneficial from both an economic and environmental perspective. However, the effect of the KR filler on the maximum crystallization rate was not linear between the PCL/3% KR and PCL/5% KR formulations, varying with the cooling rate. For high cooling rates, the highest values computed for the PCL/3% KR and PCL/5% KR were 2.394 and 2.027 min^−1^ at 20 °C/min, and 2.307 and 2.277 min^−1^ at 25 °C/min, representing the highest values at the maximum dx/dt point. This indicates that depending on the cooling rate applied, there is an inversion of behavior in the crystallization process.

**Figure 9:**
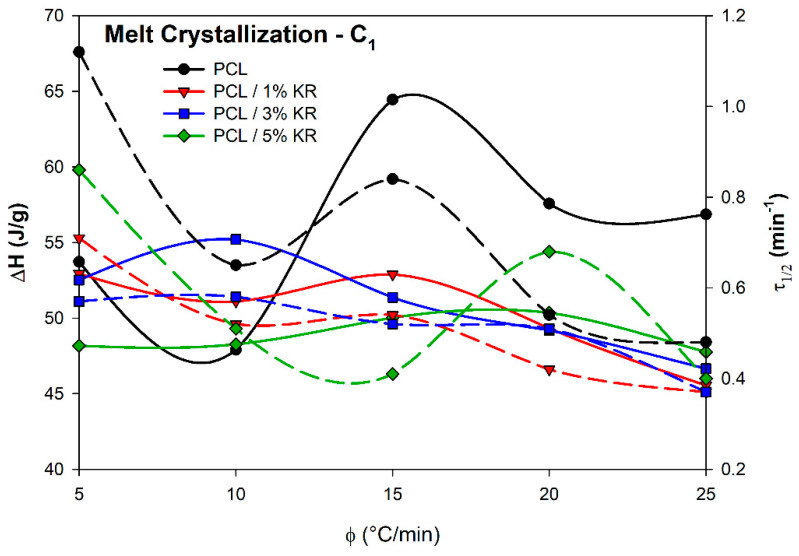
Enthalpy (solid line) and half-time crystallization (dotted line) as cooling rate function. Composites indicated.

Figure 9 shows the enthalpies (ΔH) and times to reach 50% crystallization (τ_1/2_) as functions of the cooling rate for PCL and PCL/KR composites. Regarding the ΔH of the crystallization process, oscillations were observed as a function of the cooling rate for the PCL and PCL/KR composites. However, in general, the KR filler reduced both the ΔH and τ_1/2_ due to its role as a nucleating agent that accelerates crystallization, as demonstrated in Table 1. By reducing the energy required to initiate crystallization, the process occurs more quickly, which can be advantageous in manufacturing processes.

In general, the enthalpy of the process is expected to decrease with an increasing cooling rate [52,53]. This results from insufficient time for nucleation, primary crystallization, and secondary crystallization, which hinders the organization of the lamellae and the formation of a well-ordered crystalline structure [37,54]. However, the samples exhibited an increase in ΔH from 5 to 10 °C/min, except for the PCL/1% KR, and an increase in ΔH from 10 to 15 °C/min, except for the PCL/3% KR. A decrease in ΔH was observed between the rates of 15 and 20 °C/min, except for the PCL/5% KR, which showed a decline in ΔH only at the 25 °C/min rate. These changes in enthalpic values may be linked to the crystallization mechanisms and activation energy, which will be discussed in later sections.

The PCL/3% KR formulation exhibited the best predictive behavior (in terms of ΔH and τ_1/2_), as both decreased with the increasing cooling rate (φ), suggesting it may be an optimal composition for industrial treatments. This behavior is in convergence with the good mechanical results presented by the PCL/3% KR composite, as previously verified. At higher cooling rates, such as 20 and 25 °C/min, the PCL/KR composites demonstrated a decrease in τ_1/2_ and a reduction in ΔH. The PCL/KR formulations showed very similar ΔH and τ_1/2_ values at 25 °C/min, indicating the potential for using higher kaolin residue content with this controlled cooling rate in large-scale processes.

### 2.8. First (F_1_) and Second (F_2_) Fusion Measurements

**Figure 10:**
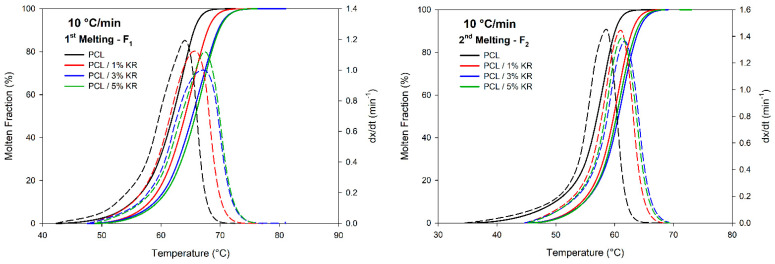
Molten fraction (F_1_ and F_2_) (solid lines) and melting rates (F_1_ and F_2_) (dotted lines) as temperature function. Composites and cooling rates indicated.

Figure 10 shows the sigmoidal curves for the first melting (F_1_) and second melting (F_2_) at a heating rate of 10 °C/min, with the results for other heating rates provided in Supplementary Material Figures S3 and S4. Table S1 presents the kinetic and thermodynamic parameters for F_1_, including the maximum melting rate (c_max_), melting onset temperature (T_1F_), peak melting temperature (T_PF_), final melting temperature (T_2F_), and enthalpy (ΔH). The subscripts in the temperatures for F_1_ and F_2_ denote the first (F) and second (S) melting stages, respectively.

In general, melting is less sensitive to heating rates and KR addition, which is evidenced by the subtle shift of the dx/dt rate peaks. During the first melt, the melt fraction, temperature, melt rate, and absolute c_max_ values were quite similar, with a slight shift to higher temperatures due to the addition of the KR. This is likely associated with the formation of larger and more perfect crystals, leading to a slight increase in the crystalline melting temperature. The computed c_max_ values during the first melt at 10 °C/min for the PCL and PCL/KR (1%, 3%, and 5%) composites were 1.193, 1.124, 1.000, and 1.124 min^−1^, respectively, without significant variations with the effect of the kaolin residue. The same trends were observed for the other heating rates, as verified in Table S1.

During the first heating cycle, PCL and PCL/KR composites may undergo thermal transitions and structural transformations, which can affect the thermal response. Therefore, the second heating cycle more accurately reflects the behavior of PCL and PCL/KR composites, offering a more stable and repeatable view of their thermal behavior. Table 2 provides the parameters for F_2_, labeled as c_max_, T_1S_, T_PS_, T_2S_, and ΔH, respectively.

Regarding F_2_, the investigated composites exhibited sigmoidal profiles and melting rate curves similar to those of F_1_ (See Figure 10). The crystal reordering that occurs during the second heating cycle promotes the formation of better ordered crystals, as reported in the literature [55,56,57]. Thus, there was an extension of the melting initiation stage compared to F_1_, although it started at lower temperatures. The addition of the KR subtly shifted the melting sigmoids to higher temperatures, without significantly affecting the maximum melting rate (c_max_) across the composites. For instance, at 10 °C/min, the maximum melting rates in increasing order of nucleating agent were 1.456, 1.448, 1.359, and 1.384 min^−1^. An increase in the fusion rate (dx/dt) was observed compared to F1, along with its occurrence at lower temperatures, suggesting that fusion is facilitated for F_2_. When PCL and PCL/KR composites are heated during the first cycle (F_1_), they may be in a state of internal stresses, resulting from deformations that occurred during manufacturing, such as in injection molding. During this initial heating cycle, these stresses tend to be relieved, causing structural changes in the PCL and PCL/KR composites. In the second cycle (F_2_), the stresses have already been relieved, and the observed thermal behavior more accurately reflects the properties of PCL and PCL/KR composites in their final state. These points will be further discussed in the context of the activation energy for fusion in the following sections.

**Figure 11:**
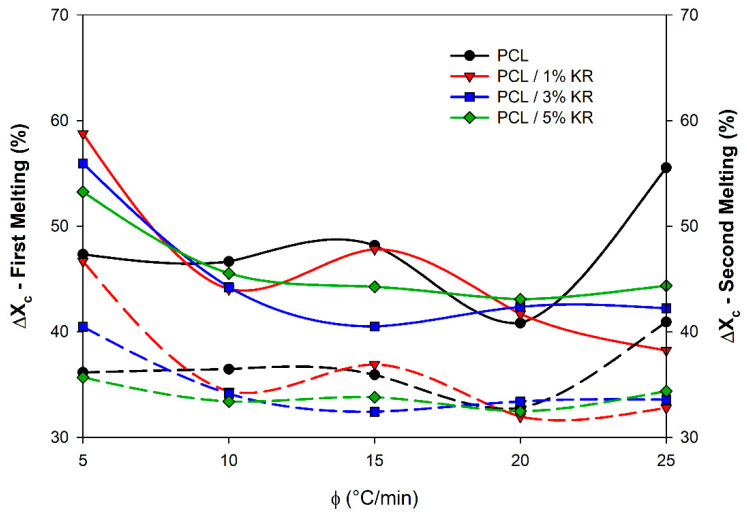
Degree of crystallinity (ΔX_c_) developed during F_1_ (solid lines) and F_2_ (dotted lines). Composites indicated.

The degrees of crystallinity calculated for F_1_ and F_2_ are presented in Figure 11 as a function of the heating rate. In general, adding the KR filler does not have a linear effect on the degree of crystallinity of the PCL matrix, with similar profiles observed for ΔX_c_ in both the first and second melting stages. Among the composites, for F_1_, the PCL/5% KR formulation exhibits higher ΔX_c_ values with increasing heating rates (except at 5 and 15 °C/min). For F_2_, the PCL/1% KR formulation shows the highest ΔX_c_ values at heating rates below 15 °C/min, while the PCL/3% KR and PCL/5% KR exhibit the highest values at 20 and 25 °C/min rates.

### 2.9. Activation Energy ($E_{a}$)—Melt Crystallization

The description and methodology for determining the energy activation are presented in the Supplemental Material (M1), with the reference base [58].

**Figure 12:**
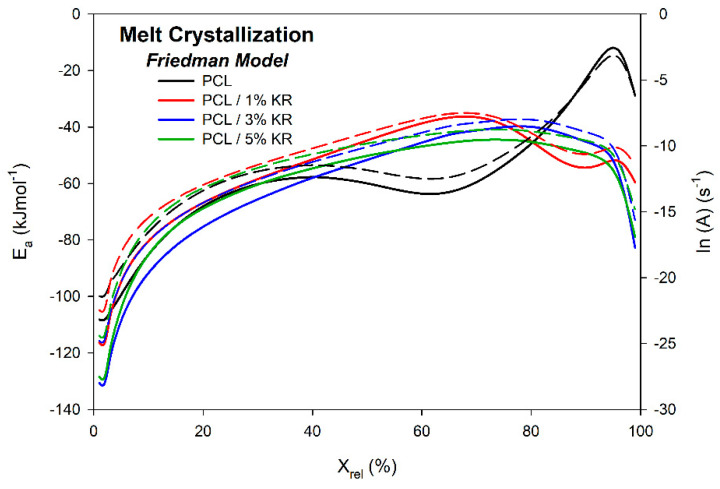
$E_{a}$ for the melt crystallization of PCL and PCL/KR composites evaluated using the Friedman isoconversional method. Compositions indicated. E_a_ (solid lines) and ln(A) (dotted lines).

Figure 12 shows the activation energy E_a_ and the frequency factor ln(A) as a function of relative crystallinity X_rel_ for all the specimens investigated in this study. All the activation energies are negative, indicating that energy must be removed from the system to promote crystallization. Considering the absolute values of the activation energies, less energy needs to be removed from the PCL/KR composites compared to the pure PCL. This suggests that the KR acted as a nucleating agent, accelerating the nucleation and crystallization process and enabling it to occur at higher temperatures than in the pure PCL. Similar trends are observed in the E_a_ graphs for the PCL/KR composites, which differ from the behavior of the PCL matrix at X_rel_ = 40%. The similar profile of the E_a_ lines indicates the occurrence of comparable crystallization mechanisms from the melt for both the PCL and PCL/KR composites. The PCL/3% KR and PCL/5% KR formulations exhibit the lowest absolute E_a_ values removed from the system, consistent with the relative crystallinity graphs, as these composites show the fastest nucleation, primary, and secondary crystallization processes. Similar energy profiles were reported by Barreto et al. [54] in their study on the crystallization of PBT/TiO_2_ composites.

The reduced energy requirement in the molding process means reduced operating costs and a more sustainable manufacturing process. In addition, accelerated crystallization reduces the time required for the PCL/KR composite to solidify, reducing the cooling time required and consequently reducing the cycle time of the manufacturing process.

### 2.10. Activation Energy ($E_{a}$)—First Fusion

When compared to crystallization kinetics, fusion kinetics have not been as widely studied, even though they are a powerful tool to confirm the trends and processing behaviors of polymers as a function of the melting conversion degree [59]. Integral or differential methods can be applied depending on the nature of the experimental data. Since the reported data are from DSC, the isoconversional model of Friedman is an efficient tool for investigating fusion kinetics and measuring the activation energy (E_a_) under heating and superheating conditions [60,61]. It is expected that the activation energy for fusion decreases as the molten fraction increases [61], due to the change in the material’s physical structure and heat diffusion through the specimen, which requires less energy with the increasing molten fraction to complete the melting process. In this work, the coefficient of determination (R^2^) for the first fusion is in the range 0.97209 ≤ R^2^ ≤ 0.99014.

**Figure 13:**
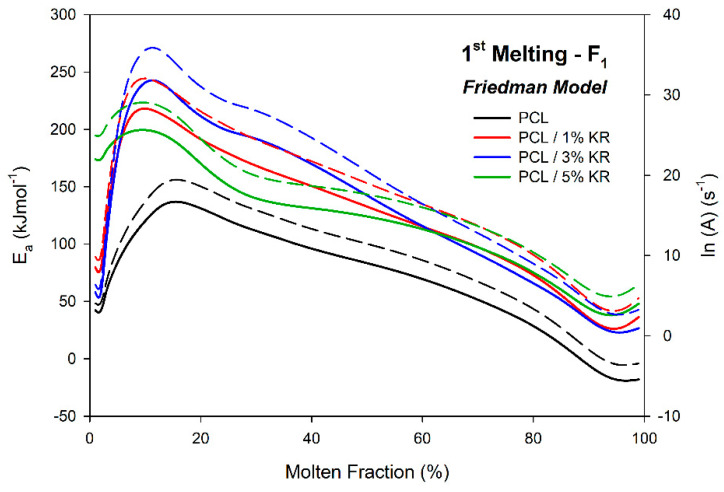
$E_{a}$ for the first melting of PCL and PCL/KR composites using the Friedman isoconversional method. Compositions indicated. E_a_ (solid lines) and ln(A) (dotted lines).

Figure 13 shows the activation energy of fusion (E_a_) and the frequency factor (ln(A)) as a function of the molten fraction for the PCL and PCL/KR specimens. All the materials showed a decrease in E_a_ within the molten fraction range of 0.1 to 0.9. The energy increase conditions between 0 and 0.1 are associated with the highest energy barriers for initiating the process and promoting phase transition. At the same time, the range between 0.9 and 1.0 is related to changes in the heat transfer mechanism from conduction to convection [62,63,64].

Regarding the E_a_ and ln(A) of the PCL/KR composites, all the formulations showed higher values for these parameters compared to the PCL matrix, results that are consistent with the melt fraction data (F_1_), where PCL is the first to undergo the phase transition by melting (indicating a lower energy barrier). Increases in Ea were observed up to a KR content of 3%. For example, at 50% melt fraction, the E_a_ values were 83, 133, 142, and 124 kJmol^−1^ in increasing order of KR filler content. The 5% KR content led to a reduction in activation energy. However, the melt fraction results in F_1_ for this formulation were significantly close to those of the 3% KR formulation, indicating a facilitated process (as evidenced by the higher maximum melting rates for 5% KR).

### 2.11. Activation Energy ($E_{a}$)—Second Fusion

**Figure 14:**
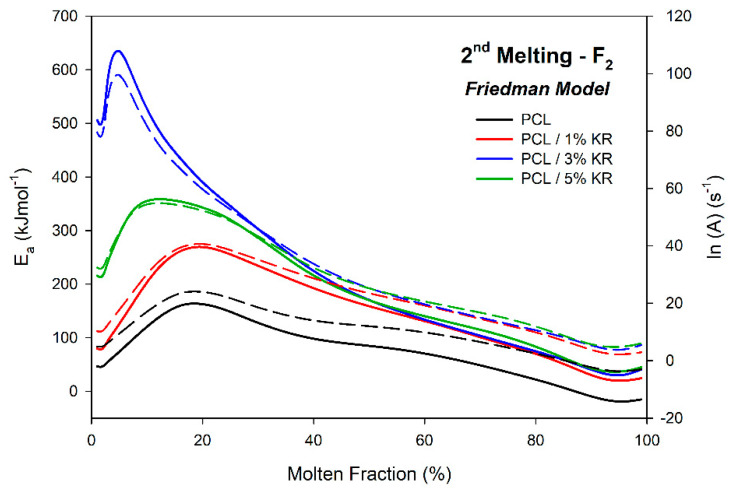
$E_{a}$ for the second melting of PCL and PCL/KR composites using the Friedman isoconversional method. Compositions indicated. E_a_ (solid lines) and ln(A) (dotted lines).

The activation energy of fusion (E_a_) and the frequency factor (ln(A)) for the second fusion (F_2_) of the investigated composites were plotted as a function of the melt fraction and are shown in Figure 14. The E_a_ and ln(A) parameters were determined using the Friedman method, similar to the approach used for F_1_. The fit of the coefficient of determination (R^2^) of the model with the experimental data is in the range 0.97681 ≤ R^2^ ≤ 0.99320.

The E_a_ and ln(A) for F_2_ showed profiles similar to those observed for F1, with some numerical differences. As seen for F_1_, the E_a_ and ln(A) values for F_2_ of the PCL/KR composites were higher than those of the pure PCL, indicating that kaolin residue in the matrix requires higher energies to drive the fusion process. As observed for F_1_, the activation energy tends to decrease with the increase in melt fraction, except for the early stages of fusion (which were extended compared to F_1_) between 0 and 0.2, and the final stages for melt fractions above 0.95.

Although the composites exhibit higher maximum melting rates in F_2_, indicating a facilitated process, the E_a_ values for F_2_ are within the same ranges as those observed for F_1_, i.e., mostly between 50 and 250 kJmol^−1^. For example, at a 50% melt fraction, the computed E_a_ values, in increasing filler order, were 84, 157, 169, and 169 kJmol^−1^, similar to those of F_1_. However, the higher maximum melting rates are attributed to the smaller temperature difference (ΔT = T2 − T1) in the F_2_ process compared to F_1_, as seen in Table S1 (Supplementary Material) and Table 2. The smaller temperature range for fusion, combined with similar energy values, results in higher event occurrence rates.

The activation energy values for the second fusion, presented in Figure 14, differ from those shown in Figure 13 for F_1_, as the first fusion is related to the material from processing. In contrast, the second fusion involves the material crystallized from the melt. This material has a distinct thermal history and morphology compared to the pre-melted material in F_1_, resulting in different Ea values [65].

### 2.12. Model-Based Kinetics Analysis

**Figure 15:**
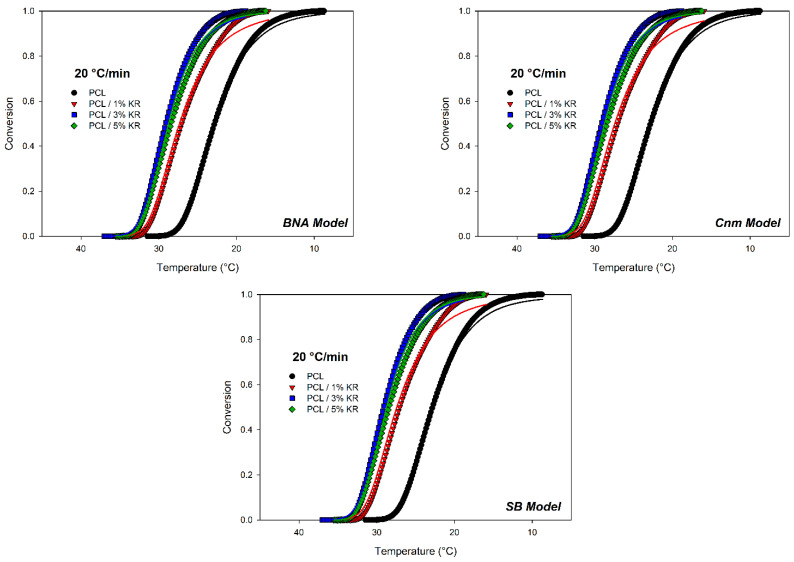
Conversion as function of temperature for PCL and PCL/KR composites under cooling rate of 20 °C/min. Applied mechanisms indicated. Symbols (experimental data) and solid line (applied model).

The kinetic analysis based on the models was performed using the NETZSCH Kinetics Neo 2.5 software package. The methods used included the expanded Prout–Tompkins equation (BNA), the nth-order reaction with m-power autocatalysis by product (Cnm), and the Sestak–Berggren (SB) equation [51,66,67]. Figure 15 shows the correlation between the experimental data (symbols) and the theoretical model fit (solid line) as a function of temperature at a cooling rate of 20 °C/min, for the BNA, Cnm, and SB models. Table 3 presents the kinetic parameters from the analysis. Figures S5–S7 in the Supplementary Material show the correlation between the experimental and theoretical data for the other cooling rates for the BNA, Cnm, and SB models, respectively.

The activation energies calculated by the Friedman differential method agree with the results obtained by model-based methods, presenting Ea values that are significantly close and all negative. The three methodologies show high coefficients of determination, with R^2^ > 0.97, and can be estimated as approximations of the characteristic function f(x) of kinetic modeling. The model-based methods agree on the order and nature of the process, with activation energies ranging from −96 to −107 kJmol^−1^. Incorporating up to 3% of KR into the PCL matrix results in Ea variations between −96.65 and −106.69 kJmol^−1^ (Cnm). The PCL/5% KR formulation shows a value of −97.5 kJmol^−1^ of energy removed from the system for the crystallization process. Similar trends were observed for the Ea calculated by Friedman.

All the frequency factors are negative, indicating that the collisions between molecules are very small, which is an expected result with the reduction in temperature and the solidification of the compound from the molten state. The Bna and SB models show similar orders, while the Cnm model showed significantly lower orders for the collision factor. The different numerical values are associated with the boundary conditions of the model and the equation for describing the same process, resulting in statistically significant values for the dx/dt approximations.

Regarding the reaction orders n and autocatalytic orders, these are as follows: PCL: n = 1.7, Autocatorder = 0.640–0.627, and m = 640; PCL/1% KR: n = 1.87, Autocatorder = 0.674–0.662, and m = 0.674; PCL/3% KR: n = 1.71, Autocatorder = 0.711–0.666, and m = 0.711; and PCL/5% KR: n = 1.56, Autocatorder = 0.678–0.688, and m = 0.678. The significantly similar values for the parameters indicate that the addition of the kaolin residue does not alter the crystallization mechanism that predominates during the primary and secondary crystallization, acting exclusively as a nucleating agent, accelerating the process, and reducing the energy barriers (increasing the efficiency in energy removal) for crystallization at higher temperatures compared to the pure PCL.

## 3. Methodology

### 3.1. Materials

Poly-(ε-caprolactone) (PCL) type CAPA^®^ 6500, was supplied by Perstorp Winning, in the form of granules, with a melt flow index of 28 g/10 min, and a molar mass of 47,500 g/mol. Kaolin residue (KR), sourced from CADAM S.A. (a fine and ultrafine kaolin production industry based in Brazil), was used as a mineral filler for the PCL. The KR used is the by-product generated during the processing of kaolin for use in the paper industry.

### 3.2. Production of PCL/KR Composites

The PCL was dried at 50 °C in an air circulation oven, while the kaolin residue was dried at 110 °C for 24 h. The kaolin powder was refined in a hammer mill and sieved to reduce its particle size, using a 200 Mesh (0.074 mm) sieve. A detailed characterization of the kaolin residue can be found in the literature [68]. The PCL/KR composites were prepared in mass ratios of 100/0%, 99/1%, 97/3%, and 95/5%. The pure PCL was processed under the same conditions as the PCL/KR composites, enabling a comparison using the same thermal cycle.

The PCL/KR concentrates were prepared using the melt intercalation method, with a high-speed mixer (Thermokinetic Homogenizer, MH-50H) (MH Equipment, São Paulo, Brazil). The equipment was operated for approximately 10 s, during which mixing and melting of the concentrates occurred due to friction. Subsequently, the PCL/KR concentrate was ground in a knife mill, producing flakes for dissolution in the extruder. This procedure was carried out to improve the level of KR distribution in the PCL matrix.

**Figure 16:**
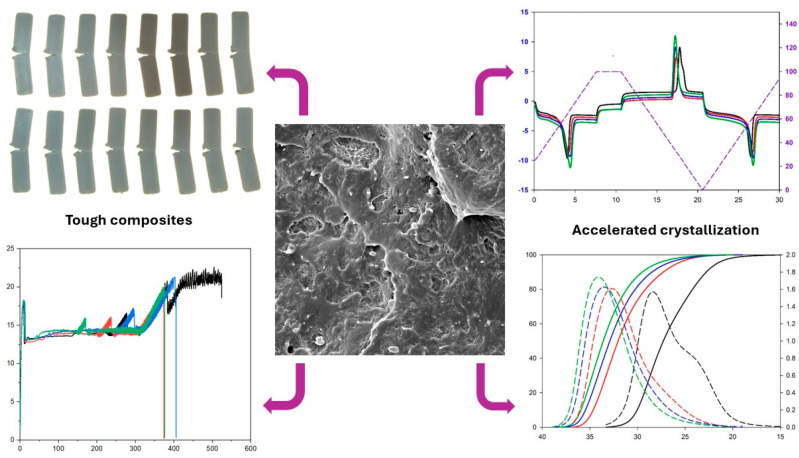
Schematic of the mechanical and thermal behavior of the samples after injection molding.

The PCL/KR composites and pure PCL were processed in a modular co-rotating twin-screw extruder, model ZSK (D = 18 mm and L/D = 40), from Coperion Werner-Pfleiderer. The temperature range used was 80 °C, 80 °C, 90 °C, 90 °C, 100 °C, 100 °C, and 100 °C, with a screw speed of 200 rpm and a controlled feed rate of 3 kg/h. Subsequently, the extruded pellets of pure PCL and the PCL/KR composites were injection molded (Arburg, Allrounder 207C Golden Edition) (Arburg Inc., Loßburg, Germany). The molding was conducted at temperatures of 100 °C, 100 °C, 100 °C, 100 °C, and 100 °C, with an injection pressure of 1000 bar, holding pressure of 600 bar, mold temperature of 20 °C, and a cooling time of 30 s. Figure 16 provides a graphical overview of the sample’s behavior following the injection molding.

### 3.3. Characterization of PCL and PCL/KR Composites

The tensile mechanical test was conducted using an Oswaldo Filizola BME universal testing machine (Oswaldo Filizola Ltda, São Paulo, Brazil), according to the ASTM D 638 standard, with a speed of 50 mm/min, and a 20 kN load cell. The notched Izod impact tests were performed using a 5.5 J pendulum on a CEAST Resil 5.5 machine (Instron, Norwood, USA), in compliance with the ASTM D 256 standard. The Shore D hardness test was carried out on a Metrotokyo machine (MetroTokyo, São Paulo, Brazil), following the ASTM D2240 standard, using a 50 N load and a 10 s stabilization time. The HDT test was performed on a CEAST (HDT 6 VICAT) machine (Instron, Norwood, USA), according to the ASTM D 648 standard, with a heating rate of 120 °C/h, a load of 455 kPa, and a deflection of 0.25 mm. Scanning electron microscopy (SEM) was conducted on the fracture surface metallized with gold, using a voltage of 20 kV, and VEGA 3 TESCAN equipment (TESCAN, Brno, Czech Republic).

The kinetic analysis of the PCL and PCL/KR composites was performed using differential scanning calorimetry (DSC) with TA Instruments DSC-Q20 apparatus (TA Instruments, New Castle, USA). The tests were conducted on samples within a temperature range of 0 to 100 °C, at heating rates of 5, 10, 15, 20, and 25 °C/min. Each analysis included a 3 min isothermal step, with a gas flow of 50 mL/min under a nitrogen atmosphere, using samples with a mass of (5 ± 1) mg. The applied kinetic modeling can be found in previous works by the authors [51,54,55,56,57,58]. The degree of crystallinity (X_c_) by DSC was determined using Equation (1):

(1) $$X_{c}=\frac{{\Delta H}_{m}}{{{\Delta H}_{0}\times W}_{PCL\;\;}\;}$$

where ΔH_m_ = the melting enthalpy determined by DSC; W_PCL_ = the mass fraction of the PCL in the composition; and ΔH_0_ = the melting enthalpy for the PCL with 100% crystallinity and 136 J/g [69].

## 4. Conclusions

The PCL/KR composites were successfully developed, demonstrating the potential of kaolin residue (KR) as an effective nucleating agent for poly(ε-caprolactone) (PCL). Incorporating 1–5% KR enhanced the elastic modulus and heat deflection temperature (HDT) of the composites compared to pure PCL, while maintaining high ductility, tensile strength, and Shore D hardness. These improvements indicate a favorable balance between stiffness and toughness, essential for practical applications. Thermal analyses confirmed that KR accelerates the crystallization process and increases the crystallization temperature. Among the compositions, the PCL/3% KR exhibited the most balanced thermal and mechanical performance. Although KR had a minimal impact on melting behavior, slight shifts in melting peaks and lower energy barriers during crystallization were observed. These findings are relevant for optimizing industrial processing, offering faster crystallization and improved thermal control. Overall, this work highlights the viability of using kaolin residue to improve both the processing and mechanical behavior of biodegradable polymers like PCL. By incorporating an abundant industrial by-product, this study supports the development of more sustainable materials and contributes to waste valorization. The addition of KR into PCL not only adds value to a low-cost residue but also aligns with environmental and economic goals, reinforcing its potential in circular economy strategies and in the production of good-performance, eco-friendly composites.

## Figures and Tables

**Table 1:** Kinetic and thermodynamic parameters of melt crystallization of PCL/KR composites.

φ (°C/min)	Parameter	PCL	PCL/1% KR	PCL/3% KR	PCL/5% KR
5	c_max_ (min^−1^) T_1C_ (°C) T_PC_ (°C) T_2C_ (°C) ΔH (J/g)	0.825 35.5 32.8 25.5 53.72	1.093 38.3 36.2 29.0 52.93	1.302 39.2 37.3 30.6 52.51	1.199 39.6 37.2 30.3 48.17
10	c_max_ (min^−1^) T_1C_ (°C) T_PC_ (°C) T_2C_ (°C) ΔH (J/g)	1.582 32.2 28.3 19.2 47.9	1.601 35.6 32.7 22.4 51.10	1.635 36.2 33.5 23.9 55.20	1.748 36.5 34.2 25.5 48.27
15	c_max_ (min^−1^) T_1C_ (°C) T_PC_ (°C) T_2C_ (°C) ΔH (J/g)	1.689 30.6 24.6 13.3 64.45	1.691 33.2 29.4 17.4 52.89	1.891 34.2 30.8 19.3 51.37	2.1359 34.7 31.9 21.1 50.02
20	c_max_ (min^−1^) T_1C_ (°C) T_PC_ (°C) T_2C_ (°C) ΔH (J/g)	1.902 27.9 23.9 13.5 57.57	1.946 32.0 28.2 16.8 49.32	2.394 33.2 29.3 20.7 49.19	2.027 33.2 29.9 18.9 50.35
25	c_max_ (min^−1^) T_1C_ (°C) T_PC_ (°C) T_2C_ (°C) ΔH (J/g)	1.818 27.4 23.5 12.7 56.86	2.191 31.9 28.6 18.7 45.55	2.307 34.5 29.9 20.3 46.65	2.277 34.5 29.8 19.7 47.76

**Table 2:** Kinetic and thermodynamic parameters of second melting of PCL/KR composites.

φ (°C/min)	Parameter	PCL	PCL/1% KR	PCL/3% KR	PCL/5% KR
5	c_max_ (min^−1^) T_1S_ (°C) T_PS_ (°C) T_2S_ (°C) ΔH (J/g)	0.988 47.8 59.6 62.9 50.23	0.898 50.4 60.0 64.7 50.66	1.005 50.6 59.8 63.7 49.91	0.975 50.6 60.3 64.7 45.55
10	c_max_ (min^−1^) T_1S_ (°C) T_PS_ (°C) T_2S_ (°C) ΔH (J/g)	1.456 47.7 58.5 64.2 64.87	1.448 50.5 61.0 67.3 47.73	1.359 50.5 61.8 68.4 51.29	1.384 50.5 61.1 67.7 44.39
15	c_max_ (min^−1^) T_1S_ (°C) T_PS_ (°C) T_2S_ (°C) ΔH (J/g)	1.632 47.9 61.2 70.4 56.23	1.537 50.2 63.2 73.1 47.41	1.604 50.2 62.8 72.7 45.05	1.741 50.38 62.1 70.9 46.40
20	c_max_ (min^−1^) T_1S_ (°C) T_PS_ (°C) T_2S_ (°C) ΔH (J/g)	1.988 51.0 62.7 74.9 49.57	1.915 51.0 63.5 74.3 46.40	1.963 51.0 63.1 74.9 46.96	1.803 51.0 64.7 76.6 45.11
25	c_max_ (min^−1^) T_1S_ (°C) T_PS_ (°C) T_2S_ (°C) ΔH (J/g)	2.128 48.8 64.5 77.5 51.59	2.247 50.5 64.1 77.5 43.94	2.066 49.3 64.2 77.5 46.12	2.449 52.4 64.8 77.5 42.88

**Table 3:** Kinetics parameters calculated for PCL and PCL/KR composites depending on the crystallization mechanism.

Mechanism Function	Model Parameters	PCL --	PCL 1% KR	PCL 3% KR	PCL 5% KR
Bna Mechanism	$E_{a}$ (kJmol^−1^) ln A (s^−1^) n AutoCatOrder R^2^	−96.65 −17.908 1.712 0.640 0.97966	−98.31 −17.846 1.886 0.674 0.97045	−106.43 −19.107 1.703 0.711 0.98559	−97.50 −17.610 1.560 0.678 0.98347
Cnm Mechanism	$E_{a}$ (kJmol^−1^) ln A (s^−1^) n m R^2^	−96.65 −27.901 1.712 0.640 0.97966	−97.247 −27.654 1.876 0.674 0.97029	−106.69 −29.150 1.705 0.711 0.98581	−97.50 −27.602 1.560 0.678 0.98347
SB Mechanism	$E_{a}$ (kJmol^−1^) ln A (s^−1^) n AutoCatOrder R^2^	−96.25 −17.844 1.698 0.627 0.97957	−96.37 −17.518 1.864 0.662 0.97010	−106.36 −19.097 1.721 0.666 0.98540	−97.52 −17.612 1.565 0.668 0.98346

## Data Availability

Data are contained within the article and Supplementary Materials.

## Supplementary Materials

The following supporting information can be downloaded at: https://www.mdpi.com/article/10.3390/ijms26104632/s1.
