# Supplementary material for: Reusing Kaolin Residue from the Mining Industry to Produce PCL-Based Composites: Accelerating the Crystallization Process and Improving Mechanical Properties
Source: Int J Mol Sci. 2025 May 13;26(10):4632. doi: 10.3390/ijms26104632 (PMC12111448; doi:10.3390/ijms26104632)
Supplement: Supplementary file 1 [file ijms-26-04632-s001.zip › ijms-3614338-supplementary.pdf]

Reusing Kaolin Residue from the Mining Industry to Produce PCL-Based Composites: Accelerating the Crystallization Process and Improving Mechanical Properties

Carlos Bruno Barreto Luna[1*], Jessika Andrade dos Santos Nogueira[1], José Vinícius Melo Barreto[2], Elieber Barros Bezerra[1], Fabiano Santana da Silva[1], Lorena Vanessa Medeiros Dantas[1], Renate Maria Ramos Wellen[2], Edcleide Maria Araújo[1]

[1]Academic Unit of Materials Engineering, Federal University of Campina Grande, Av. Aprígio Veloso, 882 - Bodocongó, 58429-900, Campina Grande - Paraíba, Brazil.

[2]Department of Materials Engineering, Federal University of Paraíba, Cidade Universitária, 58051-900, João Pessoa, PB, Brazil.

* To whom correspondence should be addressed: brunobarretodemaufcg@hotmail.com

SUPPLEMENTARY MATERIAL

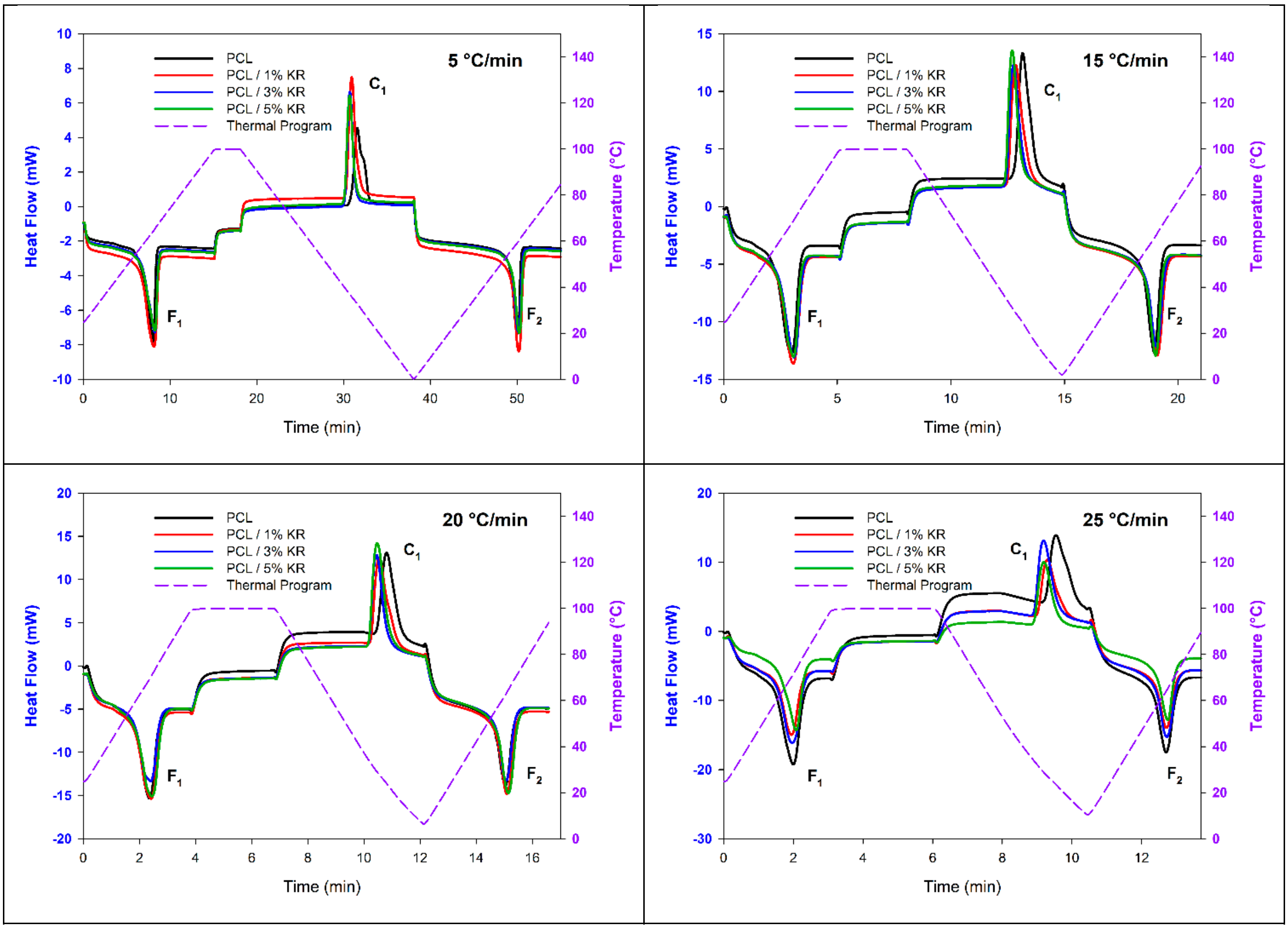

Figure S1: DSC scans for PCL and PCL/KR composites. collected during applied thermal cycles for the heating/cooling/re-heating under 5; 15; 20 and 25 °C/min. Dotted purple line is the applied thermal program.

Melt Crystallization – C_1

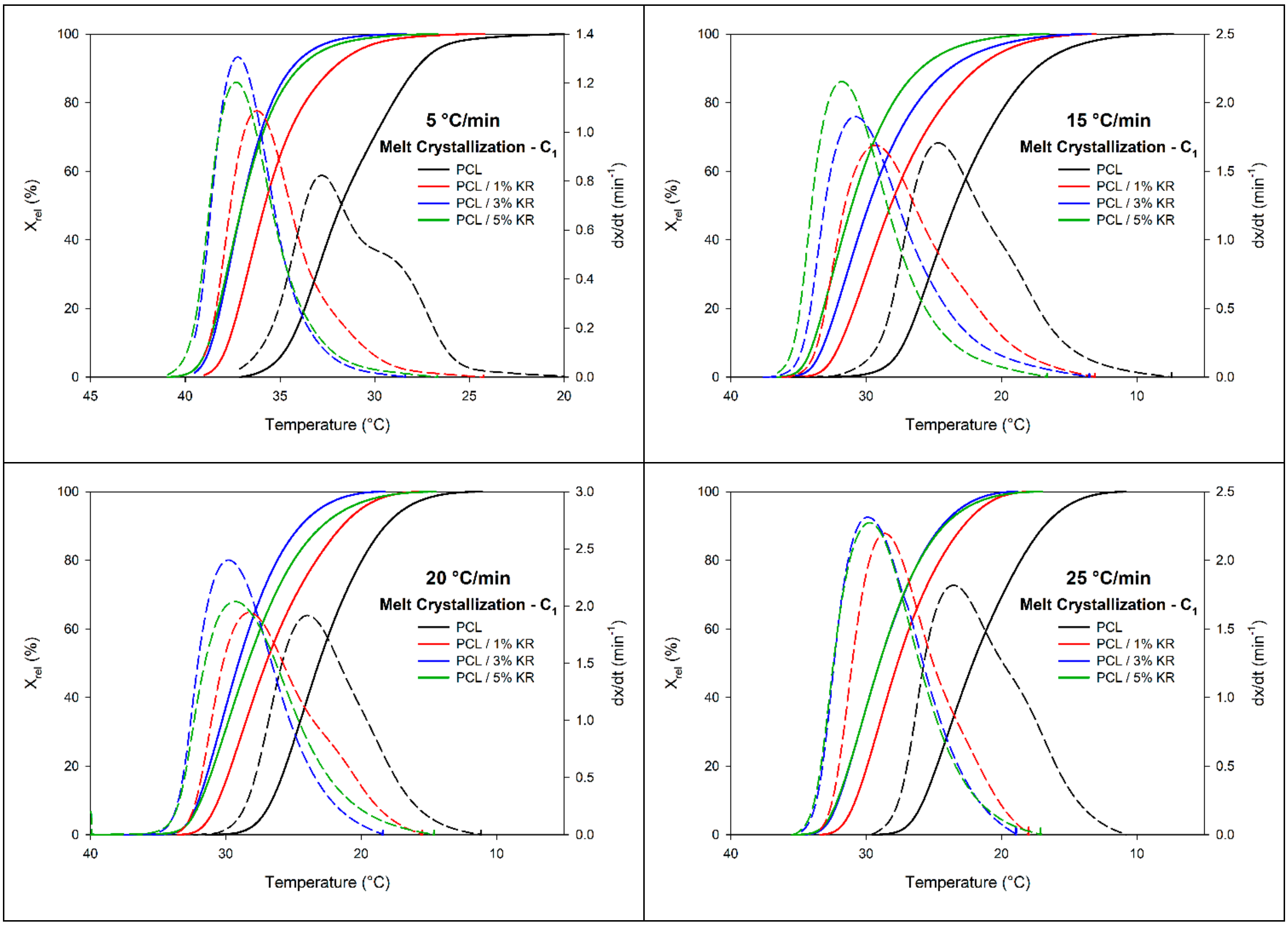

Figure S2: Relative crystalline (solid line) and Crystallization rate (dashed line) as temperature function. Compounds and cooling rates indicated (5; 15; 20 and 25 °C/min).

First Melting – F_1

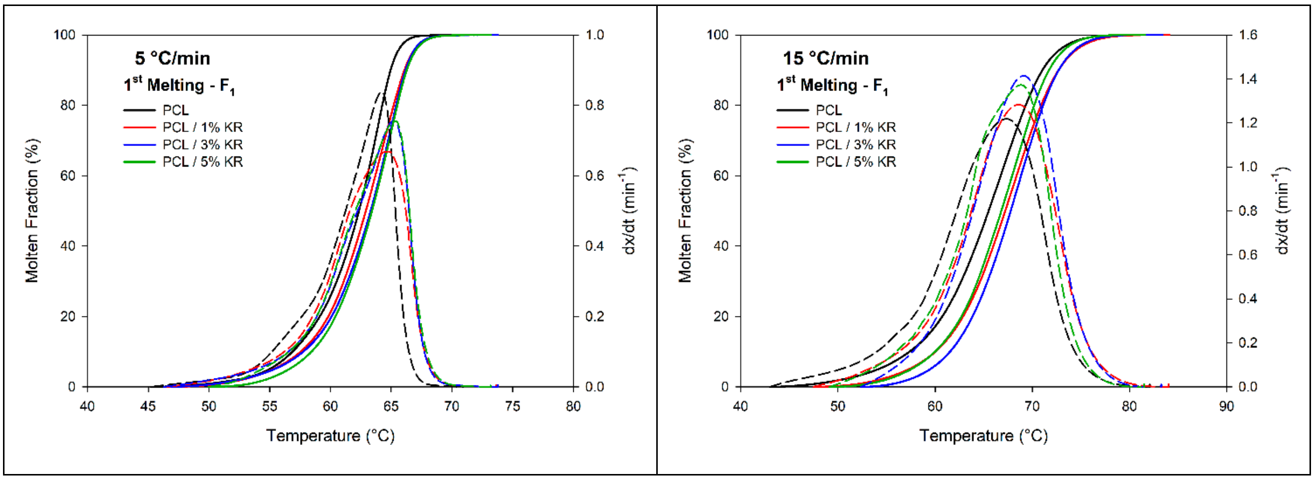

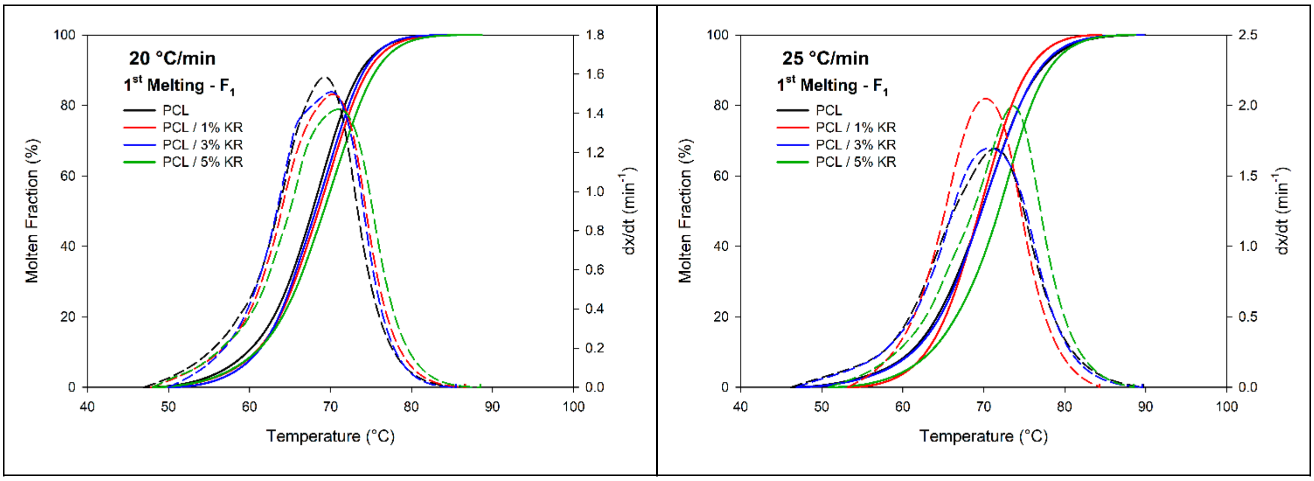

Figure S3: Molten fraction – F_1 (solid line) and Melting rates (dashed line) as temperature function. Compounds and heating rates indicated (5; 15; 20 and 25 °C/min).

Table S1. Kinetic and thermodynamic parameters of first melting of PCL/KR composites.

**φ (°C/min)**	**Parameter**	**PCL**	**PCL/1% KR**	**PCL/3% KR**	**PCL/5% KR**
**5**	c_{max} (min^{-1})	0.839	0.660	0.756	0.756
	T_{1F} (°C)	51.1	51.7	52.3	52.9
	T_{PF} (°C)	64.2	64.6	65.2	65.2
	T_{2F} (°C)	67.6	69.2	69.2	69.2
	ΔH (J/g)	65.78	64.82	66.95	56.73
**10**	c_{max} (min^{-1})	1.193	1.124	1.000	1.124
	T_{1F} (°C)	48	52.1	52.8	53.3
	T_{PF} (°C)	63.9	65.7	67.2	67.4
	T_{2F} (°C)	69.5	73.0	74.5	74.5
	ΔH (J/g)	81.67	61.20	66.46	57.93
**15**	c_{max} (min^{-1})	1.226	1.288	1.414	1.378
	T_{1F} (°C)	50.1	54.6	56.2	54.6
	T_{PF} (°C)	67.5	68.6	69.0	68.9
	T_{2F} (°C)	78.1	79.2	78.8	78.2
	ΔH (J/g)	77.75	61.47	56.28	58.85
**20**	c_{max} (min^{-1})	1.579	1.497	1.509	1.416
	T_{1F} (°C)	54.3	55.0	55.6	55.7
	T_{PF} (°C)	69.4	70.0	70.1	71.2
	T_{2F} (°C)	82.6	83.0	82.3	83.0

	ΔH (J/g)	74.02	63.27	61.50	59.87
	c_{max} (min^{-1})	1.707	2.052	1.696	2.001
	T_{1F} (°C)	54.4	57.6	56.4	57.0
**25**	T_{PF} (°C)	71.3	70.1	70.9	73.7
	T_{2F} (°C)	84.0	82.5	84.6	86.7
	ΔH (J/g)	77.19	53.12	61.47	61.64

Second Melting – F_2

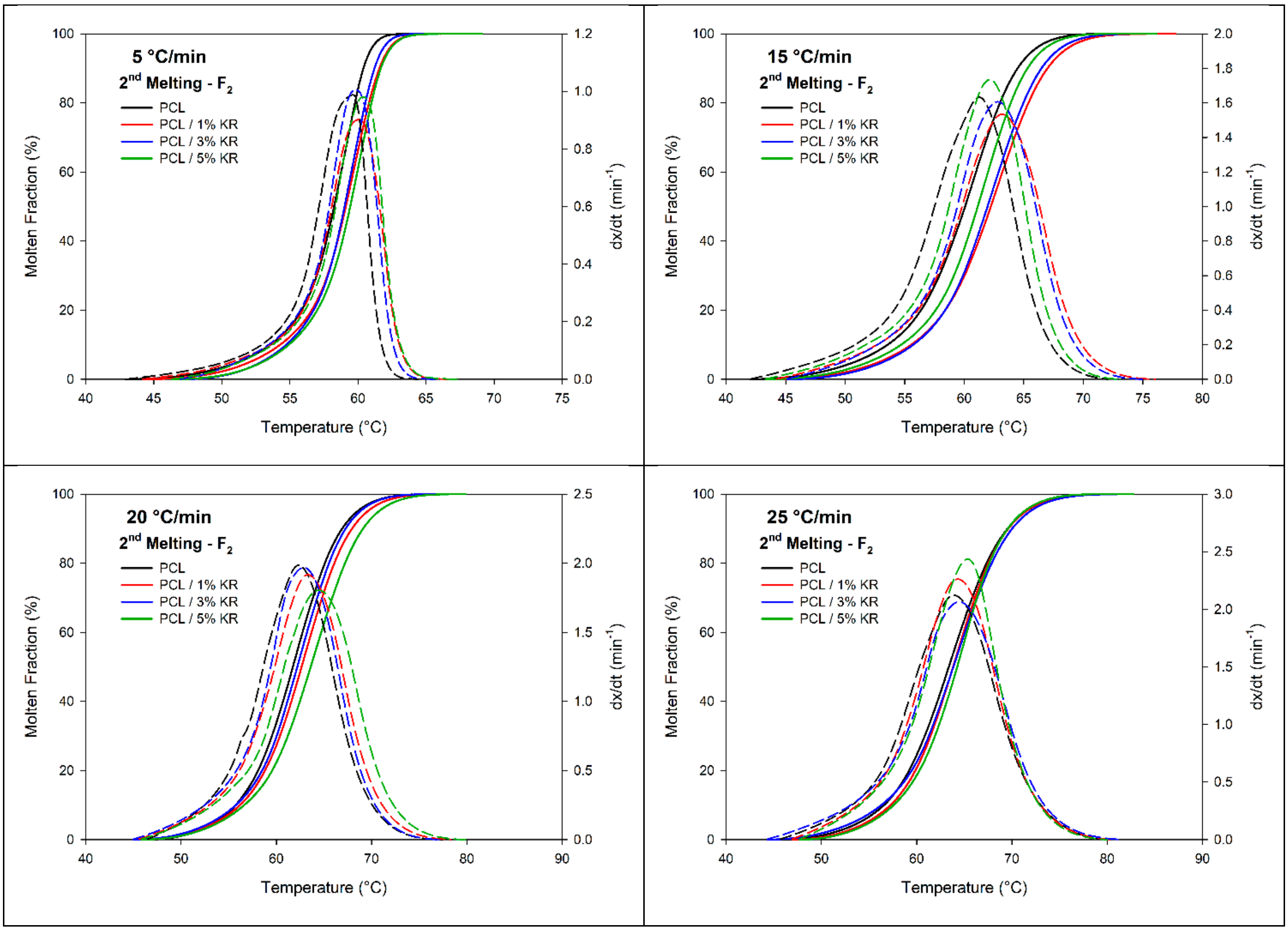

Figure S4. Molten fraction – F_2 (solid line) and Melting rates (dashed line) as temperature function. Compounds and heating rates indicated (5; 15; 20 and 25 °C/min).

Activation Energy (E_a) -M1

The conversion rate of a chemical reaction is typically expressed as the product of a temperature-dependent rate constant, k(T), and a function f(x), which represents the conversion characteristics of the reaction mechanism, as described in Eq. 1:

$$\frac{dx}{dt} = k(T)f(x) \quad \text{Eq. 1}$$

Isoconversional models are commonly used to determine the activation energy of crystallization processes. Friedman's model is based on the logarithm of the conversion rate, assuming a constant rate K(T) defined by the Arrhenius equation, as presented in Eq. 2:

$$k(T) = A\exp(-\frac{E_a}{RT}) \quad \text{Eq. 2}$$

Where A is a constant pre-exponential factor and R = 8.314 J K^{-1} mol^{-1} is the universal gas constant.

Transformations from the amorphous to the crystalline state in solids or polymeric melts are regarded as complex reactions. Therefore, Eqs. 1-2 should be generalized as follows:

$$\frac{dx}{dt} = A\exp(-\frac{E_a(x)}{RT})\, f(x) \quad \text{Eq. 3}$$

In general, E_a is a function of conversion (in this case it is a function of X_{rel}), and Eq. 3 can be converted to the logarithmic form:

$$ln\left(\frac{dx}{dt}\right) = ln\,[A.f(x)] - \frac{E_a(x)}{RT} \quad \text{Eq. 4}$$

For a given relative crystallinity x, plotting $ln\left(\frac{dx}{dt}\right)$ x $\frac{1000}{T}$, using data obtained at different cooling rates, produces a straight line with a slope of $\frac{E_a}{R}$. Repeating this analysis for various X_{rel} values yields E_a as a function of X_{rel}. The coefficient of determination (R2) for PCL and PCL/KR composites is in the range $0.97235 \leq R^2 \leq 0.98811$.

Bna Model

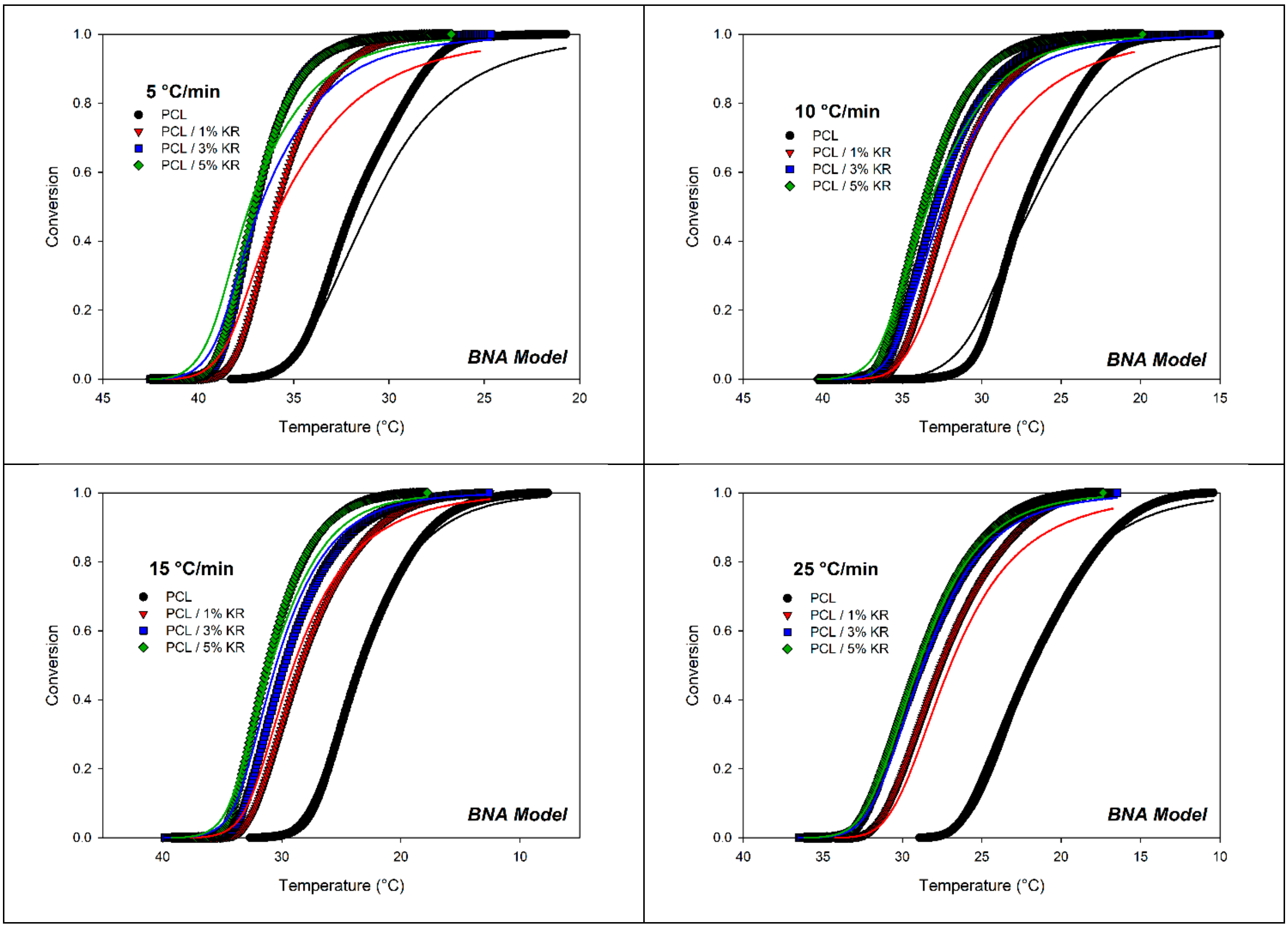

Figure S5: Conversion as function of temperature for PCL and PCL/KR composites under cooling rates of 5; 10; 15 and 25 °C/min. Applied Bna mechanism indicated.

Cnm Model

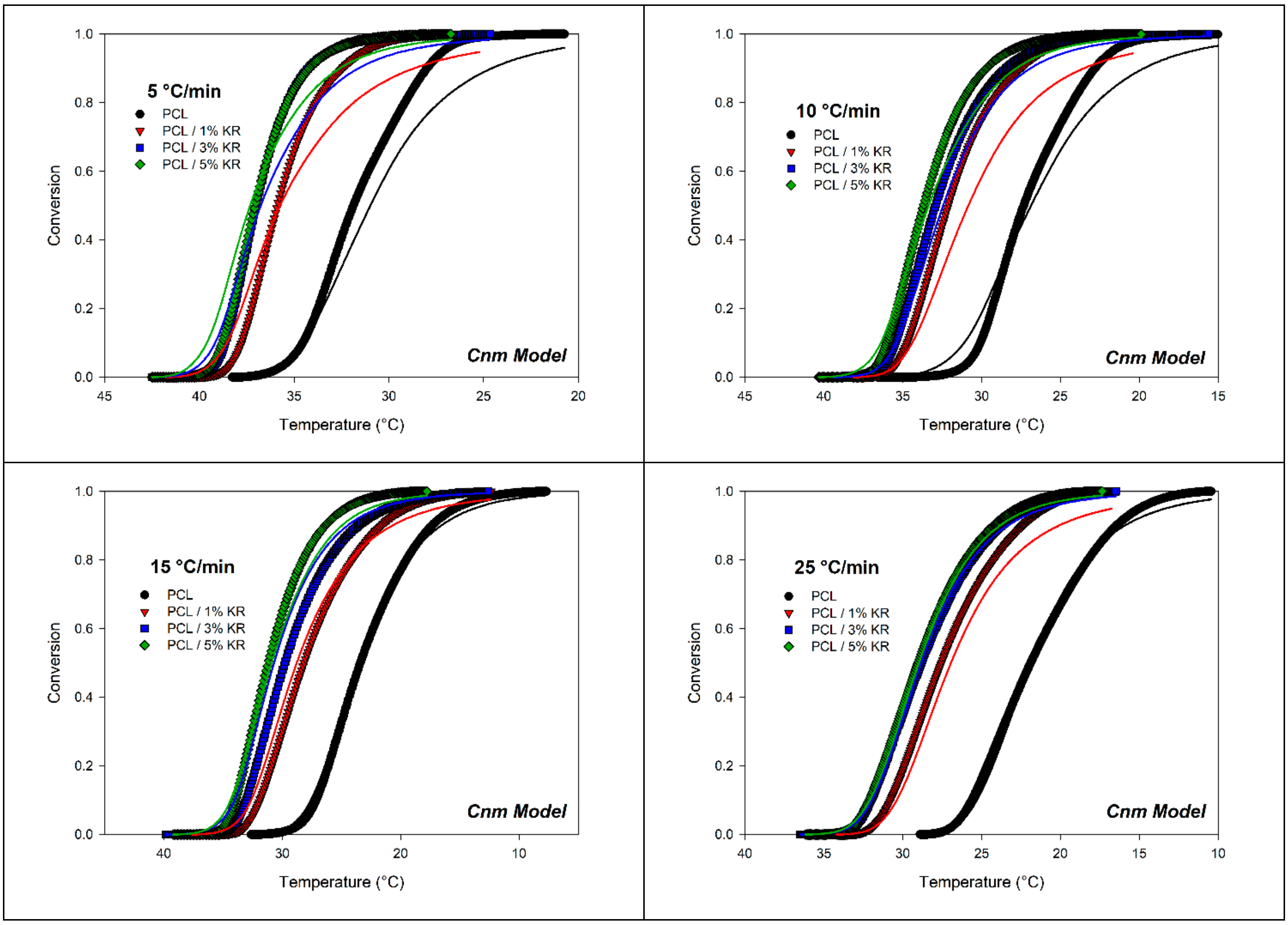

Figure S6: Conversion as function of temperature for PCL and PCL/KR composites under cooling rates of 5; 10; 15 and 25 °C/min. Applied Cnm mechanism indicated.

SB Model

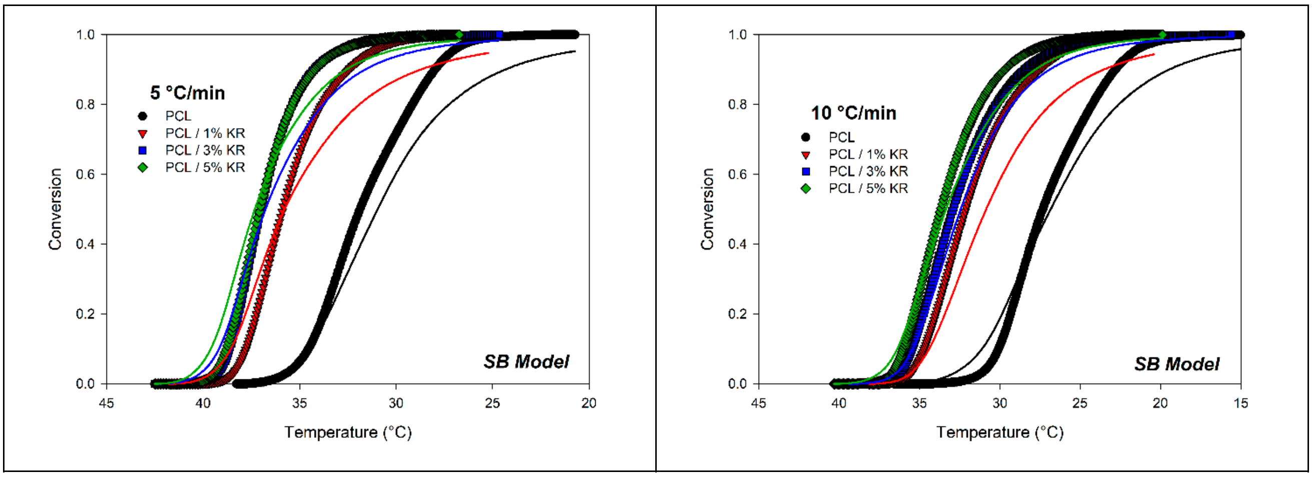

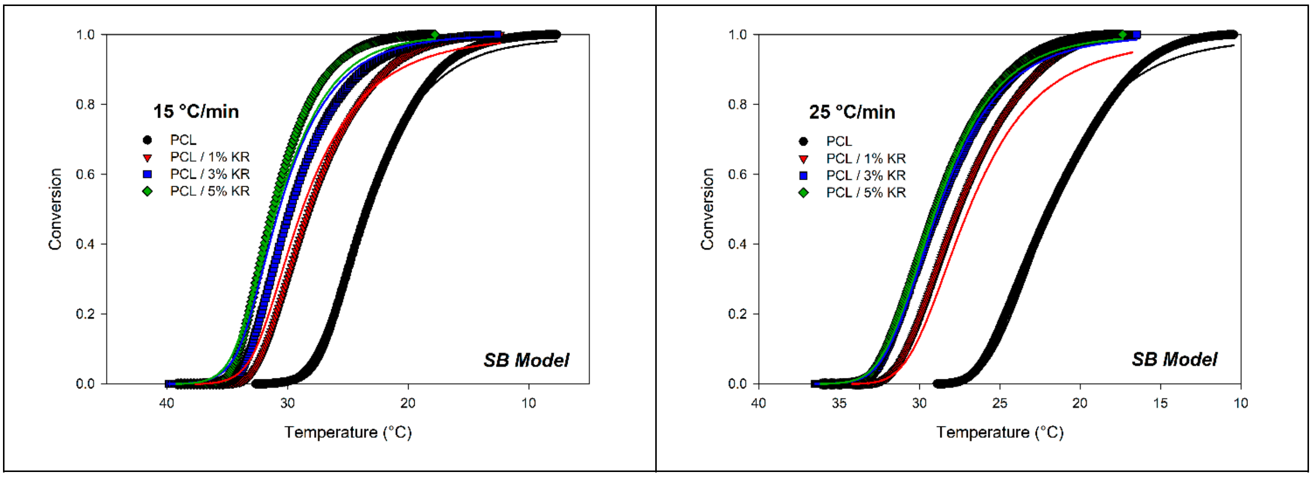

Figure S7: Conversion as function of temperature for PCL and PCL/KR composites under cooling rates of 5; 10; 15 and 25 °C/min. Applied SB mechanism indicated.
